# Sex differences in preclinical models of hypertension

**DOI:** 10.1038/s41371-022-00770-1

**Published:** 2022-11-05

**Authors:** Sol Olivera, Delyth Graham

**Affiliations:** School of Cardiovascular and Metabolic Health, University of Glasgow, 126 University Place, Glasgow, G12 8TA UK

**Keywords:** Hypertension, Hypertension

## Abstract

Hypertension remains the primary contributor in the development of cardiovascular disease which is rapidly increasing worldwide. High blood pressure affects men and women differently and understanding these sex differences is the ultimate unmet need for researchers in this field. Due to the inherent differences in hypertension prevalence, control and outcomes between men and women, novel research needs to be carried out to tackle these disparities and improve targeted treatment. Animal models of hypertension have provided valuable insights into the sexual dimorphism of blood pressure mechanisms. The availability of genetic and non-genetic hypertensive strains allows the opportunity to study diverse environmental and genetic factors that affect blood pressure, therefore presenting a valuable tool for researchers. Sex differences are present before birth and throughout life, which presents a challenge for the study of disease development in humans, but these complexities can be resolved with the use of in vivo models that display similarities to human disease. The aim of the present review is to provide an overview of the different available animal models of hypertension that present sexual dimorphisms and to discuss their relevance to humans.

## Introduction

It is estimated that more than one billion adults aged between 30 and 79 years old are currently suffering from hypertension worldwide [1], and these figures are expected to surpass 1.5 billion people by 2025 [2]. The sex-specific global burden of hypertension was approximately 1 in 5 in women and 1 in 4 in men in 2015 [3]. These differences are attributable to the sexual dimorphism in the control and regulation of blood pressure, which appears to vary by age i.e., men have higher rates of hypertension before the age of 50 compared to women, however, after menopause women are at more risk of hypertension than men [4]. Even though sex and gender differences in disease have been recognised for many decades, the number of human studies that investigate pathological differences between men and women is limited. Accounting for sex as a biological variable allows investigators to broaden the scope of their research since profound sexual dimorphisms are present in many human pathologies, including hypertension.

### Relevance of animal models of hypertension

The value of animal research in hypertension is irrefutable. Animal models are useful and versatile tools for the study of a wide range of human pathologies that allow reproducible experiments to be carried out. Hypertension is not an exception to this. Animal models of hypertension have contributed to the discovery of specific genetic loci, molecular pathways and pharmacokinetics because of their ease of use and manipulation of genotype and phenotype and, most importantly, their ability to imitate human disease. Hypertension is known to be affected by both genetic and environmental factors. Due to this multifactorial nature, different animal models have been created in order to mimic specific contributing pathways involved in the hypertension disease process, including genetic mutations, the renin-angiotensin-aldosterone system (RAAS) and elevated salt intake [5]. These variables can be controlled throughout the lives of research animals, thereby evidencing the relevance of in vivo models in this field of research.

Animal models of hypertension have provided valuable information about the mechanisms involved in the regulation of blood pressure. Rodents are mostly used for this, especially the rat which has historically been favoured over the mouse by biomedical researchers. Even though the translation from animals to humans remains a challenge nowadays, several hypertensive rat models have allowed the identification of hypertension candidate genes that have contributed to the discovery of novel blood pressure determinant loci in humans [6–10]. Despite this, candidate gene studies derived from experimental models have shown to be mostly unsuccessful due to several reasons, including the small impact of the genetic variants, poor experimental design and lack of consistency, among others [10]. Some authors argue that the utility of animal models will never be sufficient to achieve full translational capacity due to the inherent differences from species to species, and suggest that only human research would benefit clinical translation [11]. Even though the translational success rate from animal to human experiments appears to be variable [12], it can be improved by carrying out systematic reviews a priori [13]. Therefore, efforts can be made to improve the value of animal models as a source of information in hypertension research.

Similar to humans, sex differences have been reported in animal models of hypertension. In fact, the genetic mechanisms of sexual dimorphisms are now better understood due to the discoveries made through animal research. The first evidence of sex differences in blood pressure regulation of animal models was reported in normotensive dogs in 1949: male dogs exhibited higher average and median blood pressure values of up to 9 mmHg compared to females [14]. Similar differences have also been observed in fowls where higher blood pressure in male compared to female fowls is associated with increased vascular plaque formation which parallels the human phenotype [15]. Despite the ability to imitate the sexual dimorphism found in humans, these animals do not present enough benefits (capability to mimic human disease and ease of handling/manipulation) to be used routinely as models of hypertension. This is the reason why rodents are the preferred animal model of hypertension and the focus of the present review.

## Normotensive rodent models

Sexual dimorphism of blood pressure regulation is evident even in normotensive rodent strains e.g., normotensive Wistar-Kyoto rats (WKY) show a small but significantly elevated blood pressure compared to females of the same strain [16]. Sex hormones are believed to have an important role in the sexual differences in normotensive and hypertensive rats. Many experiments involving castrated/ovariectomised animals have been performed in order to control for this variable. Female WKY rats have greater endothelial cell nitric oxide (NO) bioavailability compared to male WKY rats [17]. The same effect is found in the outbred normotensive Sprague-Dawley rat strain, however, this enhanced NO bioavailability is lost in ovariectomised females, which is accompanied by an increase in blood pressure [18]. In contrast, oestrogen administration in ovariectomised Sprague-Dawley females lowered blood pressure and improved endothelial function [18]. Interestingly, androgens also appear to have a protective role in this strain. Castrated Sprague-Dawley males exhibited increased blood pressure levels and RAS activity [19], which supports the role of testosterone in the regulation of the RAS and, consequently, blood pressure. In addition, these changes returned to normal with testosterone treatment [19].

## Non-genetic rodent models of hypertension

Hypertension can be induced in normotensive rodents by the infusion of angiotensin II (Ang II), which has also shown sex differences whereby males display higher blood pressure compared to females under the same treatment [20]. The most common Ang II infusion models of hypertension are the Sprague-Dawley rat and the C57BL/6 mouse.

Ang II infusion in male and female Sprague-Dawley rats resulted in an increase in blood pressure and vascular dysfunction in males only [21]. Researchers have also observed an increase in renal damage and oxidative stress in Sprague-Dawley males compared to females under Ang II and high salt treatment [22]. This apparent female-associated protective effect is extended to the immune response to hypertension where Ang II-treated female Sprague-Dawley rats present increased levels of regulatory T cells (Tregs) whereas their male counterparts show elevated proinflammatory T cells [23]. These observations indicate that females are protected from inflammatory pathway-induced hypertension, which involves a reduced oxidative stress response.

Female-specific protection against hypertension is also present in Ang II-treated C57BL/6 mice. Chronic Ang II infusion in male and female C57BL/6 mice results in a greater blood pressure increase in the former [24], together with elevated proinflammatory cytokines and decreased NO production and glomerular filtration rate, which indicates evidence of kidney damage [25]. Gonadectomies in both sexes induced opposing effects with a blood pressure increase in females and a blood pressure drop in males [24].

## Genetic models of hypertension

### Spontaneously hypertensive rat (SHR)

The spontaneously hypertensive rat (SHR) is established as the gold-standard animal model for the study of hypertension due to its similarities to human essential hypertension [26]. This inbred model was developed by Okamoto and Aoki in 1963 through selective breeding of spontaneously hypertensive WKY rats without genetic manipulation or pharmacological intervention [27]. Similar to the rodent models previously introduced, this genetic model of hypertension demonstrates sexual dimorphism.

Hypertension and renal function were studied in intact males, castrated males and female SHR rats at a range of ages (from 7 up to 32 weeks) by Reckelhoff and colleagues [28]. Older males were found to have higher blood pressure than their intact female and castrated male counterparts. Similarly, other investigations found that castrated SHR males presented blood pressure levels similar to intact SHR females, however, this effect was reversed with testosterone administration, thereby highlighting the role of male hormones in the sexual dimorphism of SHR rats [29]. Kidney damage was greater in intact males with evidence of decreased glomerular filtration rate and increased proteinuria compared to castrated males and females, suggesting the role of androgens in hypertension and end-organ damage in the SHR model [28]. The impact of male sex hormones in hypertension was further supported by a blood pressure decrease following treatment with the androgen receptor antagonist flutamide in male SHR rats [30]. An increase in blood pressure and a decrease in pressure-natriuresis was observed in testosterone-treated ovariectomised female SHR rats, similar to the observations on intact male SHR rats [31]. Furthermore, Liu and Ely showed that besides worsening blood pressure and renal damage, testosterone increases sodium reabsorption in the kidney of ovariectomised and intact SHR females, although this effect was slightly attenuated in the latter group suggesting the protective role of oestrogen [32]. This was further investigated by the treatment of SHR females with oestrogen, which attenuated their hypertension [33]. This evidence not only suggests the important role of androgens in hypertension but also their impact on blood pressure regulation and end-organ damage, together with a potential protective role of female sex hormones in the SHR model.

Male-specific genetic predisposition appears to also have a role in sex differences in SHR hypertension. Several Y chromosome-associated loci in SHR males have been associated with the sexual dimorphism of hypertension that not only have roles in testosterone release and testis development, but have also been involved in neurohumoral activation including increased activity of the sympathetic nervous system (SNS) [34] and the regulation of the RAAS [35]. Overactivation of the SNS has been associated with the development of hypertension in SHR rats. Increased sympathetic activity was observed in SHR males containing an SHR-derived Y chromosome compared to male WKY and male SHR with a WKY-derived Y chromosome [34]. Moreover, the rise in testosterone in young male SHR rats was correlated with an increase in blood pressure in SHRs carrying the SHR Y chromosome, but not in SHR rats with a WKY-derived Y chromosome [36]. Moreover, Ely and colleagues showed that males from different rat strains carrying a SHR Y chromosome presented increased accumulation of collagen in the aorta and resistance vessels [37], which can be a risk factor for hypertension. These observations suggest that the hypertension characteristic of the male SHR strain may be partially caused by the genes located on the Y chromosome.

Experiments to increase oxidative stress with molsidomine (a NO donor and superoxide anion generator) were shown to increase blood pressure in male SHR but not male WKY rats [38]. The WKY normotensive strain presents a positive regulatory feedback mechanism of upregulation of antioxidant enzymes in response to high reactive oxygen species (ROS) production, however, this response appears to fail in male SHR rats [38]. In contrast, this method of oxidative stress increase did not have a blood pressure elevating effect in female SHR rats [39], which may be due to their elevated NOS expression and activity which can increase NO availability [40]. Blood pressure regulation in female SHR rats appears to be more dependent on NO, as chronic NOS inhibition causes a greater blood pressure increase in SHR females than in males [41]. This provides further evidence of the sex differences in the counteractive mechanisms of hypertension.

Similar to Ang II-treated Sprague-Dawley rats, there is an immune response component in the sexual dimorphism of hypertension in the SHR model. SHR rats have shown an elevated renal proinflammatory profile compared to WKYs, although female SHR rats have an improved renal anti-inflammatory mechanism compared to males [42]. It is important to note that sex hormones do not appear to account for these differences [43], suggesting that the Y chromosome-associated genes are responsible for the different mechanisms [44].

### Stroke-prone spontaneously hypertensive rat (SHRSP)

The stroke-prone spontaneously hypertensive rat (SHRSP) was established in 1974 by selective inbreeding of SHR rats displaying increased incidence of spontaneous strokes [45]. Since development, this strain has had a limited worldwide availability due to the maintenance of only small number of discrete breeding colonies and limited commercial supply. For this reason, it is less frequently used than the SHR model, however, the SHRSP rat presents a unique model of severe hypertension and cerebral stroke that closely resembles the human disease. Similar to its close relative the SHR, the SHRSP model also presents sexual dimorphism. SHRSP males have higher blood pressure and increased stroke incidence compared to females of the same strain [46], together with a worsened kidney function and inflammatory profile [47]. In contrast, some researchers such as Masineni et al., have failed to find any differences in blood pressure between males and females, however, an increased kidney damage and stroke mortality was present in the former [48], thereby showing an inherently worsened hypertensive phenotype associated with the male sex.

These sex differences in SHRSP rats have been partially attributed to a contribution of a Y chromosome-associated locus/loci to hypertension. Genetic crossing experiments between female SHRSP and male WKY rats and vice versa have shown different effects on subsequent offspring. For example, the offspring carrying the SHRSP Y chromosome (born from a SHRSP father and a WKY mother) had a higher blood pressure compared to the ones carrying the WKY Y chromosome (born from a WKY father and a SHRSP mother) [49]. Moreover, consomic strains consisting of WKY-derived Y chromosome on SHRSP genetic background and vice versa have demonstrated an increased blood pressure in rats carrying the SHRSP-derived Y chromosome compared to consomic animals with a WKY-derived Y chromosome [50].

Similar to the observations in SHR males, the SHRSP-derived Y chromosome appears to have a strong influence on blood pressure regulation. A male-specific decrease in NO bioavailability has been observed in the SHRSP model [47] but, interestingly, eNOS expression is increased, which may either indicate a compensatory mechanism for elevated blood pressure [51] or alterations that lead to increased ROS production through eNOS uncoupling [52]. In addition, SHRSP males present an even greater increase in the upregulation of eNOS compared to females, which further suggests that this mechanism is activated to compensate for the elevated hypertension found in males [51]. Furthermore, altered renal vascular function [42] and impaired intrarenal RAAS regulation [53] have been associated with the SHRSP Y chromosome lineage.

In addition, recent investigations have found an important role of T cells in the sexual dimorphism of hypertension in the SHRSP model. T-cell infiltration into vascular beds and organs can cause vascular dysfunction. Consomic strains derived from WKY and SHRSP rats have shown an increase in T-cell infiltration in the SHRSP, which was ameliorated by the introduction of the WKY-derived Y chromosome [54]. Perivascular T-cell infiltration was not different in normal WKY compared to WKY containing a SHRSP-derived Y chromosome, however, the latter presented elevated renal T-cell infiltration which possibly promoted sodium retention and, consequently, increased blood pressure [54]. Cytokine release from these infiltrated cells is thought to contribute to ROS production in the vascular endothelium [55].

### Dahl salt-sensitive (DSS) rats

Dahl salt-sensitive (DSS) rats were derived in 1962 from a selection of Sprague-Dawley rats susceptible to salt-induced hypertension [56]. DSS rats develop hypertension under a high-salt diet and present end organ damage and endothelial dysfunction, thereby representing a good model of salt-sensitive human hypertension.

DSS males present higher blood pressure compared to DSS females under the same low-salt diet, and the mechanism appears to be associated with the humoral activation of the RAAS and vasopressin [57] as well as the sex hormones [58]. Oestrogen protects against salt-sensitive hypertension in DSS females but this effect is lost after gonadectomy in contrast to ovariectomised SHR females which did not present any differences in comparison with their intact counterparts [59]. Female DSS rats also present higher levels of vasodilatory prostaglandins compared to males under both high- and low-salt diets, whereas NO production was decreased in both sexes under high-salt diet [60]. These results are unlike observations in the SHR model where only males were found to have decreased levels of NO [47]. This is evidence that different animal models of hypertension can help identify regulatory mechanisms in different types of hypertension.

Furthermore, it has been shown that testosterone treatment of neonatal DSS females triggers higher blood pressure compared to untreated females [58]. Likewise, castration of male DSS rats decreases kidney damage, blood pressure and angiotensinogen expression, although these effects were reversed by testosterone treatment [61]. Similar to male SHR rats, DSS males present an elevated sympathetic activation which is likely to contribute to their higher blood pressure levels compared to females [60]. Therefore, it is clear that there are several contributing factors involved in the protective regulatory mechanism in female DSS that are not present in male DSS, or at least they do not appear to be as effective.

## In utero programming and aging

### In utero programming

Sex differences have also been observed in the cardiovascular systems of male and female offspring developed under the same adverse intrauterine conditions, which has recently been reviewed elsewhere [62]. For example, undernutrition has been shown to affect placental size and consequently reflect on the incidence of hypertension in men but not women [63]. The mechanism by which the in utero environment programmes the offspring to develop hypertension remains unknown, however, insights have been obtained from several animal models. Sprague-Dawley dams fed on a high-saturated fat diet caused an increase in systolic and diastolic blood pressure in female but not male offspring [64]. Interestingly, endothelial dysfunction was observed in both males and females in this experiment, suggesting that other mechanisms are involved in the susceptibility of males to hypertension. In contrast, male WKY born from pregnancies affected by nutritional restriction showed an early onset of blood pressure increase together with vascular dysfunction compared to females born under the same conditions [65].

It is well-known that the offspring born from pregnancies affected by preeclampsia and/or intrauterine growth restriction (IUGR) are at increased risk of developing cardiovascular diseases later in life, although male offspring appear to have a higher susceptibility compared to females. A model of reduced uterine perfusion in pregnant Sprague-Dawley rats demonstrated that both IUGR males and female offspring developed hypertension at early stages of life, however, these were only maintained through to adulthood in males whereas females returned to normal levels [66] due to an oestrogen-related protection [67]. Interestingly, gonadectomy in adult male IUGR offspring caused a reduction in blood pressure, thereby suggesting the contribution of androgens to these developmental differences [68] possibly through a mechanism of increased sensitivity to the vasoconstrictor Ang II [69]. Moreover, markers of renal oxidative stress were discovered in IUGR males but not females [70]. Blood pressure and oxidative stress in these IUGR males were reduced by tempol treatment (a potent superoxide dismutase) [70], thus suggesting the role of ROS production in the sexual dimorphism of hypertension programming.

### Aging

Aging is associated with an increase in cardiovascular risk factors in humans and rodents. There is a decrease in testosterone in aging men which has been associated with an increase in mortality by cardiovascular disease [71]. For this reason, it has been hypothesised that testosterone supplementation may improve these conditions. In fact, testosterone supplements decreased blood pressure in old SHR males but increased blood pressure in young SHR males [72]. In contrast, testosterone administration has not shown positive results in human trials [73].

Furthermore, there is an increase in hypertension prevalence in post-menopausal women and aging female rats. Sixteen-month old SHR females have demonstrated increased blood pressure as well as decreased levels of oestrogen- and oestrogen receptor-mediated relaxation compared to twelve-week old SHR females [74]. Moreover, twelve-month old female DSS rats were found to have increased renal vessel stiffness and fibrosis compared to their four-month old counterparts under a low-salt diet [75]. These effects were attenuated with oestradiol supplementation which evidences the protective role of oestrogen and the increase in kidney inflammation and attenuation of eNOS levels as a result of age-associated oestrogen decrease [75]. In contrast, hormone replacement therapy does not ameliorate blood pressure in post-menopausal women [76], which suggests that oestrogen depletion is not the only risk factor in the development of hypertension in women after menopause.

## Relevance and comparison to humans

The sexual dimorphism of hypertension observed in preclinical models (Table 1) shows important similarities to the situation in humans, whereby hypertension risk factors and treatment affect men and women differently, even from a young age [77]. Blood pressure monitoring in children and adolescents between 10 and 18 years old showed increased values in boys compared to girls of the same age [78]. This difference is further maintained in young adults whose blood pressure levels are up to 6% higher in men compared to women [79]. It is important to note that these marked differences diminish after menopause: women have an increase in blood pressure which peaks at >70 years-old when the prevalence of hypertension is higher compared to men [80]. Hence, the ability of women to maintain lower blood pressure levels is lost with age and, consequently, hypertension develops rapidly [81].

**Table 1:** Summary of sexual dimorphism and hormone-dependent differences in preclinical models of hypertension.

Strain/Model	Description of sex differences	Possible molecular mechanisms
Normotensive models		
Dog	**↑** BP in males [14]	
Fowl	**↑** BP and vascular plaque in males [15]	
WKY rat	**↑** BP in males [16] **↑** NO bioavailability in females [17]	
Sprague-Dawley rat	**↑** BP in males ↑ NO bioavailability in females [18]	Sex hormones affecting RAS [19]
Non-genetic hypertensive models		
Sprague-Dawley rat (+Ang II infusion)	**↑** BP ↑ vascular dysfunction in males [21] **↑** Renal damage **↑** oxidative stress in males under high salt diet [22]	**↑** Tregs in females and **↑** proinflammatory T cells in males [23]
C57BL/6 mice (+Ang II infusion)	**↑** BP [24] **↓** NO production **↑** proinflammatory cytokines and↓ GFR in males [25]	
Genetic hypertensive models		
SHR	**↑**BP ↑ proteinuria and **↓** GFR in males [28] **↑** NO bioavailability in females? [40]	Sex hormones [28, 29] SHR-derived Y chromosome loci [34, 35] Increased vascular collagen accumulation in males [37] Altered NO signalling [40] Improved renal anti-inflammatory mechanism in females [42]
SHRSP	**↑**BP ↑ stroke incidence [46] **↑** inflammation and **↑** kidney dysfunction in males [47, 48] **↓** NO bioavailability in males [47]	SHRSP-derived Y chromosome loci [49, 50] **↑** inflammatory state in males [54, 55] Impaired NO-induced relaxation in males [47]
DSS rat	**↑** BP ↑ kidney damage [61]	Sex hormones [58, 59, 61] **↑** Sympathetic activation in males [57, 60] and **↑** vasodilatory prostaglandins in females [60]
Hormone-induced PCOS rodent models		
(EV)-induced PCOS rat model [83]	**↑** BP compared to female controls [83]	**↑** Sympathetic activity in PCOS females [83]
Peripubertal testosterone exposure mouse model [84]	**↑** BP and **↑** dyslipidaemia compared to female controls [84]	
Testosterone enanthate-induced rat model [85]	**↑** BP compared to female controls [85]	Oxidative stress dysregulation in PCOS females [85]

These results suggest that male sex hormones may play a negative role in the development of hypertension in men, as the increase in blood pressure occurs at the same time as the androgen production. This is supported by investigations in various animal models that have confirmed the important role of sex hormones through castration/ovariectomy experiments. Moreover, women with increased levels of testosterone e.g., women with polycystic ovary syndrome (PCOS) have an increased prevalence of cardiovascular disease risk factors, including high blood pressure and metabolic abnormalities, compared to healthy women [82], which further highlights the role of male hormones in hypertension and supports the results of testosterone administration experiments in female and castrated male animal models. Relevant in vivo models of PCOS evidencing the role of sex hormones in the development of hypertension include the oestradiol valerate (EV)-induced PCOS rat model [83], the peripubertal testosterone exposure mouse model [84], and the testosterone enanthate-induced rat model [85]. Each of these models demonstrates elevated blood pressure alongside other cardiovascular complications (Table 1).

Furthermore, there is a strong correlation between male blood pressure profile and paternal blood pressure status, suggesting the important role of paternal inheritance in blood pressure incidence and supporting the correlation between hypertension and the Y chromosome observed in preclinical models. In addition, the X chromosome has also been associated with sex-specific blood pressure effects in both rodents [86] and humans [87], although this link is usually masked by the protective effect of female sex hormones. Human studies have confirmed the strong genetic relationship between the Y chromosome and the development of cardiovascular disease in men [88], although investigations on the association between Y chromosome haplogroups and hypertension have yielded ambiguous results. Recent evidence suggests that several Y chromosome haplogroups in men are involved in the adaptive immune response and autoimmunity which may present shared pathways between human and rodents [44], although the association with hypertension remains to be determined. Deciphering the implications of the human Y chromosome in hypertension is challenging due to its haploid nature, however, an approach involving both rodent and human phenotypic and gene expression data may provide relevant insights [89].

Finally, stress and the activation of the sympathetic nervous system have been implicated in the development of hypertension in humans and, as observed in the SHR model, these risk factors affect men and women differently. Sympathetic nerve activity is positively correlated with cardiac output and peripheral resistance in young men, however, this relationship is not present in young women [90], thereby evidencing the differences in blood pressure regulation by gender.

Altogether, the parallel findings of sex differences between humans and preclinical models of hypertension provide evidence of the relevance of animal studies in the field of hypertension research. There is still controversy regarding the positive impact of animal research, but in reality, preclinical models have allowed the discovery of successful antihypertensive treatments and strategies, including renin [91] and angiotensin-converting enzyme (ACE) inhibitors [92], and have opened the door to the possibility of novel pharmacological interventions. Thus, the utility of the discoveries on the sexual dimorphism of hypertension observed in animals and humans can be extended into the development of new sex-specific treatment strategies. Investigations on the sex differences of antihypertensive treatments in the SHR model have demonstrated that the ACE inhibitor losartan is more effective in females than in males [93]. Even though these observations have recently been confirmed in humans [94], the prescribed frequency of this drug remains the same for both genders. This is one of the many examples of different outcomes in men and women after antihypertensive treatment, and yet there is still no difference in treatment guidelines according to gender. The influence of sex on the effectiveness of antihypertensive drugs has been documented for decades, however, there is not sufficient evidence to inform therapy. Nevertheless, it is expected that the recent discoveries made through animal research will be able to translate into improved gender-focused treatment strategies in the near future.

## Conclusion

Sex differences are the primary unanswered question in the field of hypertension research. The sexual dimorphism in hypertension appears to be evident even from early development which may determine regulatory mechanisms and susceptibility to disease through young age and into adulthood and aging. For this reason, sex is a biological variable that must not be ignored when establishing and utilising preclinical models of hypertension. The molecular basis for the sex differences in hypertension appears to rely on genetic predisposition (e.g., Y chromosome), RAAS dysregulation, oxidative stress and inflammation. These discoveries have been possible due to the research carried out in animal models of hypertension that have allowed considerable progress to be made in the field.
